# Anatomical Determinants of Tracheal Breathing Sounds: A Computational Study of Airway Narrowing and Obstructive Sleep Apnea

**DOI:** 10.3390/diagnostics15243108

**Published:** 2025-12-07

**Authors:** Walid Ashraf, Jeffrey J. Fredberg, Zahra Moussavi

**Affiliations:** 1 Biomedical Engineering Program, University of Manitoba, Winnipeg, MB R3T 2N2, Canada; zahra.moussavi@umanitoba.ca; 2 Department of Environmental Health, Harvard T.H. Chan School of Public Health, Boston, MA 02115, USA; 3 Department of Electrical and Computer Engineering, University of Manitoba, Winnipeg, MB R3T 2N2, Canada

**Keywords:** tracheal breathing sounds, obstructive sleep apnea, aeroacoustics, airway modelling

## Abstract

**Background**: Tracheal breathing sounds (TBS) have demonstrated strong potential as a non-invasive, wakefulness-based diagnostic tool for obstructive sleep apnea (OSA); yet the relationship between specific upper airway anatomical features and the resulting TBS spectra remains insufficiently understood. This study aims to enhance the diagnostic utility of TBS in OSA by investigating how the upper airway anatomy influences TBS spectral characteristics. **Method**: Patient-specific computational models of the upper airway were reconstructed from high-resolution CT scans of a healthy subject and an individual with OSA. Additional variants were generated with targeted constrictions at the velopharynx, oropharynx, and trachea, based on clinically reported anatomical ranges. Airflow dynamics were simulated using Large Eddy Simulation (LES), and the resulting acoustic responses were computed via Lighthill’s acoustic analogy within a hybrid aero-acoustic framework. **Results**: Oropharyngeal constriction generated the most spatially concentrated vorticity patterns among single-region constricted models. Airway Resistance analysis revealed that severe velopharyngeal and oropharyngeal constrictions contributed most to regional airway resistance. Spectral analysis showed that velopharyngeal narrowing produced a progressive downward shift in the third resonance peak (1000–1700 Hz), while oropharyngeal narrowing induced an upward shift of the third peak and a downward shift of the fourth peak (1700–2500 Hz). These frequency shifts were attributed to the effective role of acoustic mass and airway compliance. **Conclusions**: Anatomical modifications of the upper airway produce region-specific changes in both flow and acoustic responses. These findings support the use of TBS spectral analysis for non-invasive localization of airway obstructions in OSA.

## 1. Introduction

### 1.1. TBS Relevance

Breathing sounds remain an important part of a constellation of methods used to diagnose respiratory disorders including asthma, chronic obstructive pulmonary disease (COPD), and obstructive sleep apnea (OSA) [1,2,3,4]. These sounds arise from the interaction between airflow and anatomical structures in the respiratory tract. Among various types of respiratory sounds, tracheal breathing sounds (TBS) have gained particular interest due to their dependence upon central and upper airway (UA) morphology and their potential to detect airflow abnormalities within these anatomical regions.

OSA is characterized by recurrent airway collapse during sleep. Despite its high prevalence and significant health implications, OSA remains significantly underdiagnosed [5,6]. While overnight polysomnography (PSG) remains the current gold standard for OSA diagnosis, PSG is expensive, not readily available and resource-intensive; it requires specialized equipment, overnight patient monitoring and trained personnel. Consequently, these demands result in long wait times and limited feasibility in acute care settings such as intensive care units (ICUs) or prior to surgeries requiring general anesthesia [7,8]. These shortcomings underscore the growing need for non-invasive, accessible, and cost-effective alternatives for screening and diagnosis. In this context, analysis of TBS recorded in few minutes during wakefulness presents a promising diagnostic tool. TBS can be recorded non-invasively at the suprasternal notch, offering the possibility of detecting anatomical and functional abnormalities and thus lowering the need for PSG studies [9,10]. Unlike lung sounds, which are often attenuated by surrounding tissues, TBS are less filtered and more directly influenced by structural changes in the UA. This fact makes them particularly suitable for identifying airway constrictions and flow disturbances that are central to disorders like OSA.

### 1.2. Anatomical Determinants

The diagnostic utility of TBS arises from their sensitivity to biomechanical parameters such as local airflow velocity, wall compliance, and airflow obstruction. When air moves through a narrowed or an irregular structure within the airway, local pressure and velocity fluctuations produce acoustic features, such as amplitude changes and shifts in resonant frequencies. Importantly, these acoustic features vary systematically with anatomical and physiological alterations [11]. This sensitivity to geometry makes TBS a promising tool for identifying the severity and location of obstructions in conditions such as OSA. Structural narrowing, particularly in the velopharyngeal and oropharyngeal regions, has been observed to persist even during wakefulness, thus influencing the acoustic response and offering diagnostic insights [12,13,14].

Airway geometry plays a central role in the pathophysiology of OSA. Several imaging studies have shown that variability in airway geometry during wakefulness, such as reduced cross-sectional area [15,16], increased airway collapsibility [17,18], and altered airway shape [13] are strong predictors of airway collapse during sleep. A significant correlation was found between the UA length and the respiratory disturbance index, a metric used to assess OSA severity [19]. Similar trends were observed when the UA length was normalized to body height, further supporting the role of airway elongation in OSA pathogenesis [20]. Additionally, the cross-sectional shape of the velopharynx differs between OSA patients and healthy individuals, tending to be more circular in OSA patients and more elliptical in healthy participants during sleep [17]. Individuals with OSA also exhibit increased pharyngeal length, a thicker posterior pharyngeal wall and reduced pharyngeal width, collectively contributing to a more constricted velopharyngeal segment [13]. Smaller cross-sectional areas in the oropharyngeal and nasopharyngeal regions were reported in individuals with OSA compared to those in healthy controls [18]. Consequently, researchers investigated the relationship between the UA geometry and TBS. For instance, the increase in inspiratory sounds’ intensity from upright to supine posture was higher in OSA than in healthy individuals [10]. An inverse relationship was reported between body height, which is associated with tracheal length [21], and the resonance frequencies of TBS [22,23]. In patients with tracheal stenosis, TBS amplitude near 1 kHz was reported to be elevated compared to that in healthy controls, highlighting the sensitivity of TBS to localized UA constrictions [24].

### 1.3. Rationale for TBS Modelling

Despite these anatomical insights, the use of TBS in clinical diagnosis remains limited due to lack of mechanistic understanding of how specific anatomical features influence the acoustic response. Most diagnostic applications rely on empirical signal processing methods with limited connection between the measured sounds and the underlying physiology. Bridging this gap requires a mechanistic approach that links anatomy to breathing sound generation. Computational modelling has emerged as a powerful and non-invasive tool in respiratory physiology and biomedical engineering. By combining computational fluid dynamics (CFD) to capture airflow patterns and turbulence, with acoustic finite element modelling (FEM) to capture resulting acoustic radiation, insights might be obtained that are not accessible through clinical and experimental measurements alone. Such modelling enables the quantification of key physical variables such as pressure gradients, turbulent kinetic energy (TKE), wall shear stress (WSS), and local velocity distributions, which are essential for identifying aero-acoustic source mechanisms [11]. These variables can be spatially mapped and correlated with specific airway geometries, providing a framework for linking anatomical features to airflow dynamics and acoustic biomarkers. Moreover, aero-acoustic modelling facilitates parametric studies where individual anatomical features can be systematically varied, isolating their impact on the resulting sound signature, a benefit not feasible in vivo.

In this study, we aimed to address the research gap, namely, the incomplete understanding of how the UA anatomical traits affect the acoustic spectral features of TBS. Here, we investigate the effect of various anatomical features, such as oropharyngeal and tracheal constrictions, airway length and their interactions, on TBS using a one-way coupled aero-acoustic simulation modelling approach applied to anatomically realistic UA models reconstructed from CT scans of both a healthy individual and an individual with OSA. Previous work on TBS-based OSA assessment has primarily relied on empirical signal processing and statistical or machine-learning models [25,26] without explicit mechanistic modelling of how regional airway anatomy shapes the sound spectrum. In parallel, several computational models of the UA have examined the impact of anatomy on airflow dynamics [27] and on breathing or snoring sounds [28,29]. However, these studies have either focused on specific structures (e.g., the glottis in [28]) or on snoring-related acoustics, and the specific influence of velopharyngeal, oropharyngeal, and tracheal geometry on tracheal breathing sounds has remained largely unexplored. In this context, the present study provides, to the best of our knowledge, the first patient-specific computational framework that systematically quantifies the resulting changes in tracheal breathing sound spectra, thereby establishing a mechanistic link between localized anatomical features and TBS characteristics.

## 2. Materials and Methods

**Figure 1:**
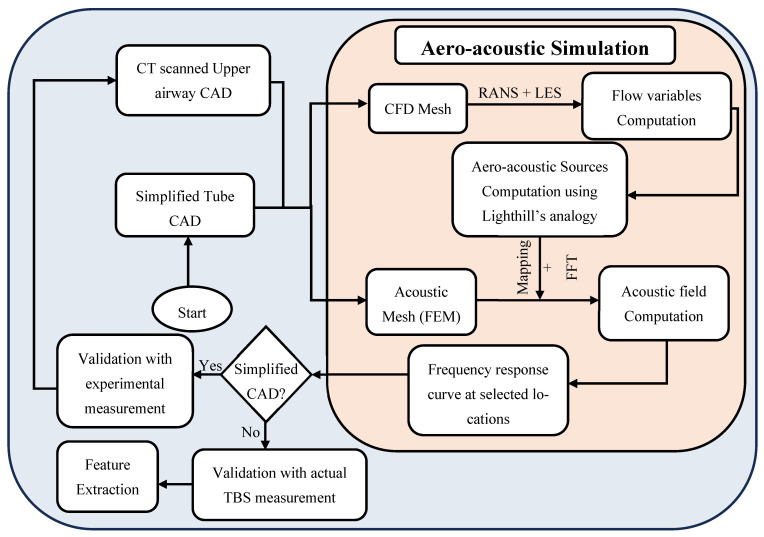
Flow Chart of the methodology procedures, based on the work presented in [11].

An overview of the methodology employed is illustrated in Figure 1. Part of the workflow, including the aero-acoustic simulation framework, CFD computation, and acoustic Finite Element Modelling (FEM), has been described and validated in our prior work [11]. Section 2.1 summarizes these previously validated components. In this study, we build upon that foundation by extending the analysis to examine the influence of specific anatomical features, including oropharyngeal and tracheal narrowing, airway length, and their interactions, on tracheal breathing sounds (TBS). The new contributions, detailed in Section 2.2, focus on integrating anatomical modifications into the simulation pipeline, validating their effects through subject-specific geometries. Feature extraction was performed to explore the diagnostic relevance of these findings in distinguishing between healthy and OSA airways.

**Figure 2:**
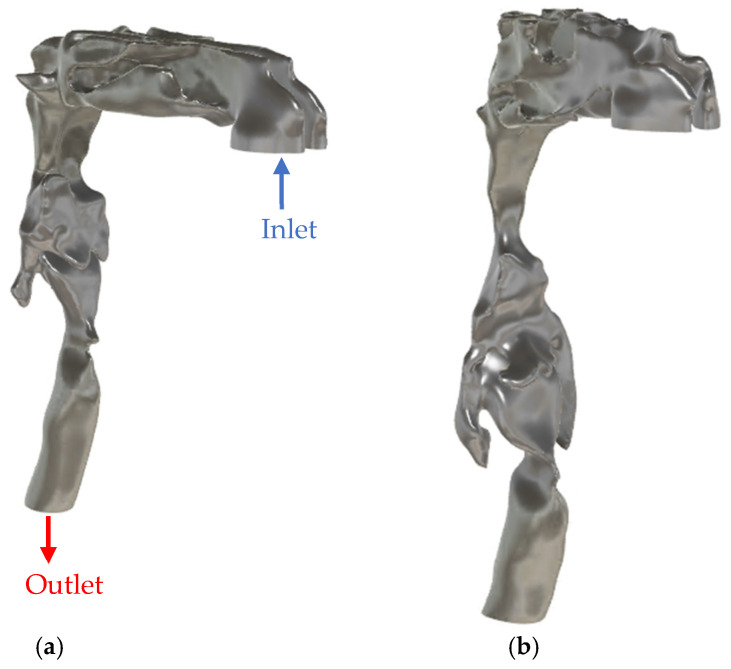
CAD model of the upper airway of (**a**) healthy participant and (**b**) OSA participant.

This study was approved by the Biomedical Research Ethics Board of the University of Manitoba [code: B2022:226 (HS25583), date: 14 July 2022]. Subject-specific airway geometries were reconstructed from high-resolution CT scans of two participants: a healthy 76Y female (height: 146 cm, BMI: 27.2 kg/m^2^) with no reported respiratory disorder and a 47Y male (height: 165 cm, BMI = 28.7 kg/m^2^) with clinically diagnosed severe OSA. The individual with OSA exhibited an apnea-hypopnea index (AHI) of 58 events per hour (85.2 events/hour in supine position). The CT volumes had a matrix size of 512 × 512 × 1057 voxels and a voxel size of 0.4375 × 0.4375 × 0.335 mm^3^. The age difference between the healthy and OSA participants may introduce potential confounding due to age-related changes in soft tissues and airway dimensions; this is acknowledged and further discussed in the limitations section. Semi-automatic segmentation using ITK-SNAP [30] was applied to reconstruct the 3D UA geometries from each scan. Post-processing was performed to ensure anatomical accuracy and mesh quality, using Autodesk Fusion 360 for gap filling and Blender for wall smoothing and inlet/outlet preparation. Surface smoothing was performed to remove stair-step and aliasing artifacts while maintaining changes in minimum cross-sectional areas in each anatomical segment below 2% and in total airway volume below 1% relative to the original segmentation. Figure 2 shows the CAD geometries for the airway of both participants.

### 2.1. Aero-Acoustic Simulation Framework

Aero-acoustic simulations can be performed using either a direct or hybrid approach. In this study, a hybrid method based on Lighthill’s analogy [31] was employed due to its computational efficiency and suitability for low Mach number flows [32] such as those observed in human respiration. The hybrid approach decomposes the simulation into two sequential steps. The first step involves a steady state Reynolds-Averaged Navier–Stokes (RANS) simulation, which is used to initialize the flow field and provide stable initial conditions, followed by unsteady, incompressible, scale-resolving finite volume CFD using large eddy simulation (LES) to capture the flow field and turbulent structures responsible for sound generation. Smagorinsky–Lilly subgrid-scale model was employed to model the effect of unresolved small-scale turbulence [33,34]. In the second step, the aero-acoustic source terms, derived from the CFD simulation, are mapped onto a coarser finite element model (FEM), where the acoustic wave propagation is computed in the frequency domain by solving the variational form of the wave equation. This modelling approach relies on several assumptions; first, the flow is incompressible and isentropic, which is valid for low Mach number flow (Ma < 0.2), and therefore, the convection effects can be neglected; second the influence of acoustic waves on the flow field is negligible, justifying the one-way coupling; third the acoustic source is compact relative to the wavelength of interest.

A pressure drop of 2.8 cm H_2_O between the inlet (nostrils) and outlet (trachea) of the UA was applied to simulate the plateau of peak inspiratory flow. This was implemented by setting the nostrils (inlet) to atmospheric pressure and applying a subglottic pressure of −2.8 cm H_2_O at the tracheal outlet [11]. This value is consistent with the reported range of inspiratory pressure [35] and, in the present study, resulted in an inspiratory flow rate of approximately 33 L/min which is inline with flow rates commonly used in the literature [36,37]. This setup was chosen to align with the validation study presented in [11], where the acoustic analysis was based on a stationary 300 ms segment of tracheal breathing sounds (TBS) corresponding to maximum inspiratory effort. The CFD simulation was performed using ANSYS-Fluent (2024-R2) with a fixed time step of 0.0001 s for 8000-time steps. A no-slip boundary condition was imposed on the airway walls. The CFD mesh consisted of approximately 2.9 M elements for the healthy model and 3.6 M elements for the OSA model. Boundary layer refinement ensured that wall-adjacent cells maintained y⁺ < 1 for accurate near-wall resolution. Mesh quality was assessed using cell skewness and orthogonal quality. The average skewness was 0.28 (95% of the cells <0.50) for the healthy model and 0.27 (95% of the cells <0.52) for the OSA model, while the average orthogonal quality was 0.70 and 0.73, respectively. The unsteady velocity fluctuations obtained from LES CFD were transformed into frequency domain and mapped onto the finite element mesh for acoustic analysis in ACTRAN-Acoustics software (2024.1). The acoustic domain was discretized using second order (quadratic) tetrahedral elements, ensuring a minimum of 45 elements per acoustic wavelength, enabling accurate sound propagation modelling up to 2500 Hz. Infinite elements [38] were assigned to the inlet surface to simulate anechoic conditions and prevent non-physical reflections. At the tracheal outlet, a normalized impedance boundary condition was imposed to approximate realistic boundary loading. To account for tissue damping and sound absorption, a normalized surface impedance of 500 (relative to air) was applied to the airway walls. The microphone probe for recording tracheal breathing sounds in the simulation was placed in the extended tracheal propagation domain downstream of the tracheal outlet, matching the position used in the experimental validation [11]. The full simulation framework, including solver configurations, governing equations, meshing and boundary conditions, has been described in detail in our previous work [11].

### 2.2. Anatomical Feature Extraction

To investigate the anatomical determinants of TBS, a series of anatomical variations were introduced into the UA geometries. These variations targeted specific regions, namely the velopharynx, oropharynx, and trachea, and were guided by reported ranges of airway dimensions for OSA patients in previous clinical and imaging studies. For instance, velopharyngeal minimum cross-sectional area (CSA) was reported as 35.3 ± 23.5 mm^2^ (mean ± std) in 17 OSA patients during wakefulness [17], while oropharyngeal minimum CSA was reported as 203 ± 95.3 mm^2^ in 20 OSA adults [14]; and 61.45 ± 45.03 mm^2^ in 33 OSA children [39]. Although pharyngeal narrowing is a well-documented characteristic of OSA, recent evidence also highlights the role of tracheal constriction in the disorder manifestation and severity [40,41]. In this study, each anatomical region was altered to simulate two levels of constriction: mild and severe. For instance, velopharyngeal constriction reduced the CSA from 85 mm^2^ (healthy) to 62 mm^2^ (−27%) for mild narrowing and to 26 mm^2^ (−69%) for severe narrowing. Oropharyngeal constrictions resulted in CSA reductions from 190 mm^2^ to 85 mm^2^ (−55%) and 30 mm^2^ (−84%) for mild and severe conditions, respectively. Tracheal constriction decreased CSA from 166 mm^2^ to 67 mm^2^ (−60%) and 36 mm^2^ (−78%) for mild and severe cases. Additionally, tracheal length was extended to reflect inter-individual anatomical variability: mild elongation increased length from 135 mm to 155 mm (+15%), while severe elongation extended it to 175 mm (+30%). These constrictions were imposed manually on the airway surface in Blender by locally narrowing the target segment; after each modification, the minimum CSA was measured on cross-sectional planes, and the geometry was iteratively adjusted until the desired percentage reduction was obtained. The selected “mild” and “severe” percentages were not based on a single fixed percentile but were tuned to produce anatomically plausible CSAs that lie within the ranges reported in OSA imaging studies and to generate clearly distinct moderate versus pronounced narrowing scenarios. The effects of velopharyngeal narrowing were investigated in prior work [11] and are included here for comparative purposes. All modifications were implemented on the CT-based healthy airway model to isolate the acoustic impact of each anatomical feature while preserving overall boundary conditions and flow dynamics. In addition to the primitive modifications applied to individual regions, several combined configurations were developed to examine the interactive effects of multilevel airway alterations. This framework resulted in a total of 15 UA configurations added to the original geometry. A complete summary of the anatomical modifications, including targeted regions, constriction severity, and geometric variations, is provided in Table 1. In addition, the minimum cross-sectional area and segmented volume of each targeted airway region of the primitive models and OSA model are summarized in Table 2.

**Figure 3:**
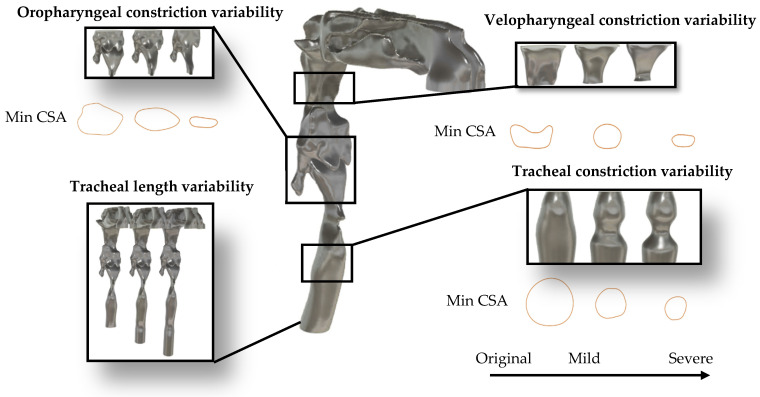
Anatomical modifications applied to the healthy airway model. Zoomed-in views show constriction and length-extension variants for the velopharynx, oropharynx, and trachea, used to simulate patient-specific anatomical changes based on OSA-related variability.

Figure 3 illustrates the anatomical variations described above. It displays the healthy CT-based airway model alongside region-specific zoomed-in views for the velopharynx, oropharynx, and trachea. Each panel illustrates the geometric variations corresponding to mild and severe constriction levels, as well as the progressive elongation of the trachea. Furthermore, to evaluate the clinical relevance of the observed acoustic changes, a simulation based on the CT scan of the OSA participant will be conducted [42]. This serves as a verification step to assess whether the parametric variations introduced in the modified models reflect physiologically realistic acoustic behavior in OSA conditions.

## 3. Results

### 3.1. Aerodynamics

**Figure 4:**
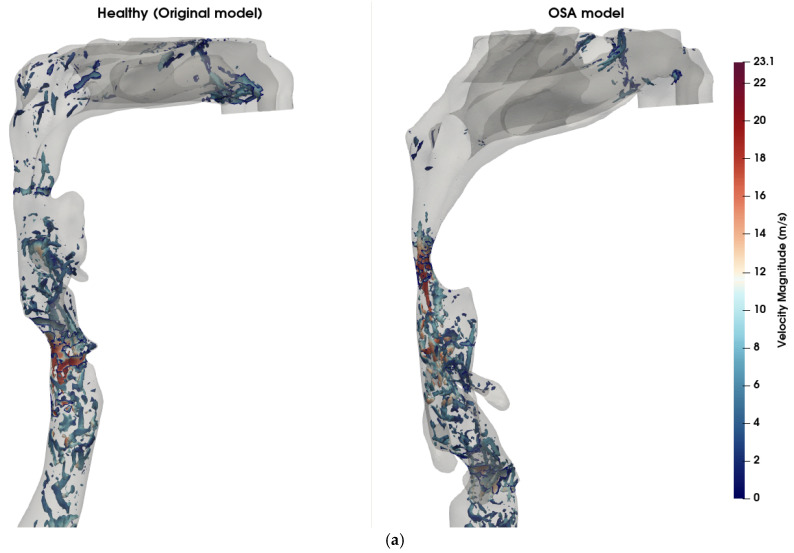

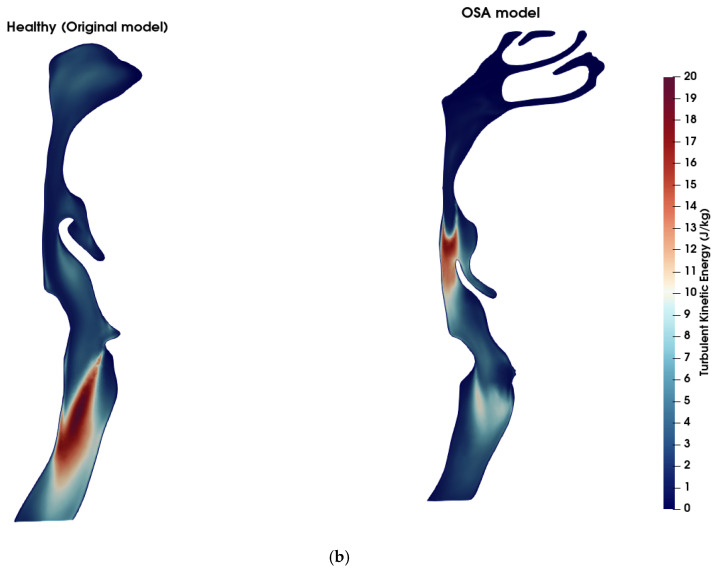
Comparison of flow features between healthy and OSA upper airway models. (**a**) Coherent vortex structures visualized using iso-surfaces of the Q-criterion (Q = 2 × 10^7^ s^−2^), overlaid and colored by velocity magnitude (m/s) at t = 0.8 s. In the OSA model (**right**), elevated velocity regions and dense vortical structures are visible near the pharyngeal constriction, indicating increased shear and potential transition to turbulence. In contrast, the healthy model (**left**) exhibits fewer and weaker vortex structures, signifying more stable and laminar flow. (**b**) Turbulent kinetic energy (TKE) contours across the airway mid-sagittal plane. The OSA model displays higher TKE values concentrated downstream of the constriction site, demonstrating enhanced turbulence generation due to velopharyngeal narrowing. The healthy model shows lower and more uniformly distributed TKE levels.

Figure 4 illustrates a comparison of airflow dynamics within the UA, emphasizing differences in vortical structures (Figure 4a) and turbulent kinetic energy (TKE) distribution (Figure 4b) between the healthy (original) model and the obstructive sleep apnea (OSA) model at t = 0.8 s. In the OSA model, a pronounced anatomical narrowing is observed in the velopharyngeal region, with ~80% reduction in cross-sectional area (CSA) at the point of minimum constriction. The vortical structures are visualized using iso-surfaces of Q-criterion at 2 × 10^7^ s^−2^, colored by local velocity magnitude. The Q-criterion isolates regions where the rotational component of the flow dominates over the strain rate, thereby highlighting areas of vortex formation. The OSA model exhibits intense and dense vortex cores, clearly observed in and downstream of the velopharyngeal constriction, corresponding to strong shear layers and the development of jet-like turbulent flow and downstream recirculation zones. These structures are associated with elevated velocity magnitude, reaching 23.1 m/s, indicating localized flow acceleration and transition to turbulence due to the anatomical obstruction. This further indicates the increase in the quadrupole sound sources at the constriction zone [43]. Although the dipole sources, associated with unsteady wall pressure fluctuations, are typically more dominant in low Mach number flows [44], the presence of intense vortical structures suggests that quadrupole sources may also contribute to sound generation in pathological cases like OSA. In contrast, the healthy model exhibits significantly fewer and weaker vortex structures in the velopharyngeal zone, indicative of a predominantly laminar flow regime. The maximum TKE in the velopharyngeal region increased from approximately 7.9 J/kg in the healthy model to 19.5 J/kg in the OSA model (≈2.5-fold increase), and the volume-average TKE within this region rose from 1.5 J/kg to 4.7 J/kg (≈3-fold increase). The TKE distribution (Figure 4b) further supports these observations, with the OSA model exhibiting higher energy levels in the velopharyngeal region. This elevated TKE reflects the conversion of mean flow energy into turbulent fluctuations, which can also modulate the acoustic signature of the UA.

**Figure 5:**
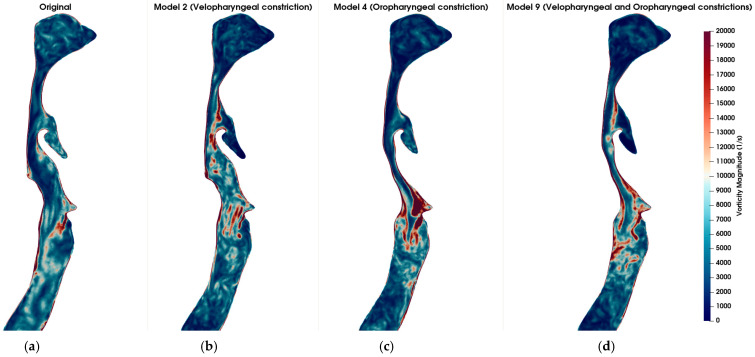

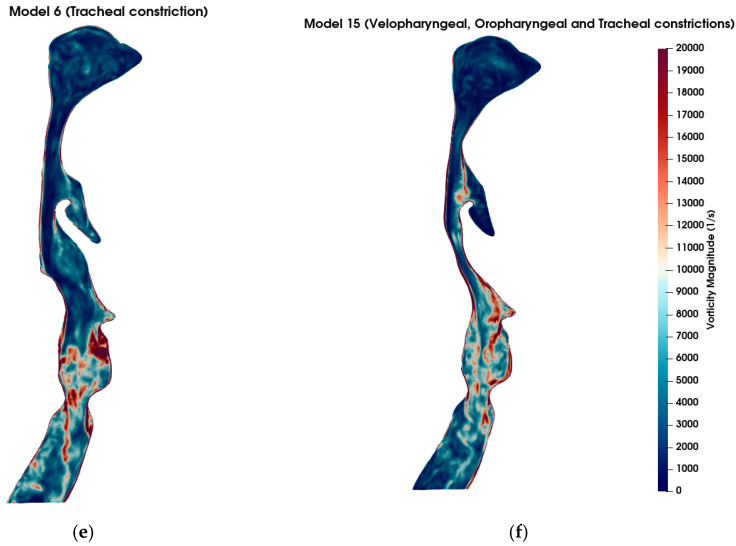
Comparison of vorticity magnitude (in s^−1^) across different airway geometries under inspiratory flow conditions at t = 0.8 s. (**a**) Original (healthy) model; (**b**) Model 2: Velopharyngeal constriction; (**c**) Model 4: Oropharyngeal constriction; (**d**) Model 9: Combined velopharyngeal and oropharyngeal constrictions; (**e**) Model 6: Tracheal constriction; (**f**) Model 15: Combined velopharyngeal, oropharyngeal, and tracheal constrictions. Vorticity magnitude fields are shown in sagittal plane slices to highlight the regions of high rotational flow associated with anatomical narrowing. The color scale is consistent across all subplots, with red indicating stronger vortical structures.

Figure 5 visualizes the vorticity magnitude in the mid-sagittal plane across six airway models: the healthy original model (a), model 2 with severe velopharyngeal constriction (b), model 4 with severe oropharyngeal constriction (c), model 9 with severe velopharyngeal and oropharyngeal constrictions (d), model 6 with severe tracheal constriction (e), and model 15 with severe velopharyngeal, oropharyngeal and tracheal constrictions (f). The vorticity magnitude captures the rotational intensity of the flow and is indicative of shear layer development and turbulence onset. In the healthy original model, mild vortex shedding is observed near the glottis region. In contrast, the models with geometric narrowing display intensified vorticity, especially downstream of the constricted zones, indicating the formation of shear-driven instabilities. Notably, model 4 (oropharyngeal constriction) exhibits the most intense and spatially concentrated vortex structures among the single-constriction models. This suggests that oropharyngeal narrowing may introduce more severe aerodynamic disturbances compared to other individual constrictions. Furthermore, in models with multiple constrictions (models 9 and 15), complex vortex interactions and elevated rotational intensity are evident along extended regions of the airway. However, it is notable that the magnitude and spatial concentration of vorticity induced by a single severe constriction exceeded those observed in models featuring multiple combined constrictions. This finding suggests that a singular severe constriction may lead to more pronounced aerodynamic disturbances characterized by stronger shear layers, intensified localized vortex shedding, and increased turbulence compared to scenarios involving several constrictions distributed along the airway. Consequently, the presence of a single, critically narrowed region may result in more severe aerodynamic conditions, potentially elevating the risk of pathophysiological consequences such as increased airway resistance, turbulent kinetic energy, and susceptibility to airway collapse typically associated with OSA.

### 3.2. Airway Resistance

**Figure 6:**
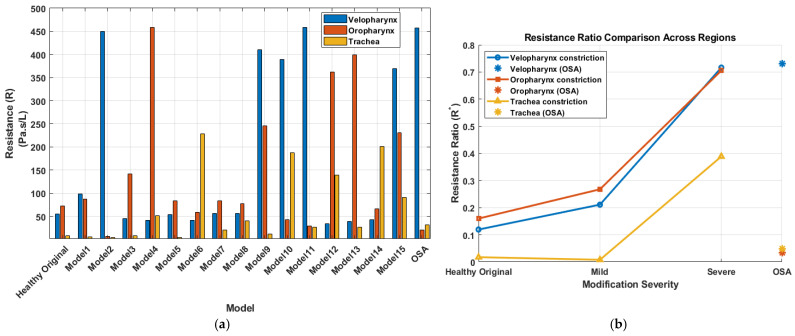
Resistance characteristics across models. (**a**) Airway resistance (Pa·s/L) for the velopharynx, oropharynx, and trachea across all models, including the original and OSA geometries. (**b**) Resistance ratio (R*) progression with increasing constriction severity (Original → Mild → Severe) for each anatomical region, with OSA resistance ratios shown for comparison.

The airway resistance was computed in the three targeted regions (velopharynx, oropharynx and trachea) for each model using Equation (1):

(1) $$R_{region}=\frac{{∆P}_{region}}{Q}$$

where $R_{region}$ is the airway resistance in Pa·s/L, ${∆P}_{region}$ is the pressure drop across the region of interest in Pa, Q is the volumetric flow rate in L/s. The velopharynx was defined from a plane through the posterior edge of the hard palate to a plane through the tip of the uvula, the oropharynx extended from the uvular plane to a plane through the superior margin of the epiglottis, and the tracheal segment was defined from a plane just below the glottis to the distal tracheal outlet from the glottis. A dimensionless resistance (R*) was also calculated to quantify the contribution of each targeted region to the overall airway resistance. This ratio was defined as the regional resistance divided by the global airway resistance, where the global airway resistance was computed using the total pressure drop across the airway (from the inlet at the nostrils to the tracheal outlet). Equivalently, this dimensionless ratio also represents the proportion of the total pressure drop occurring across each individual region. Figure 6a shows the airway resistance computed across the three anatomical regions of interest, for all airway models studied, including the healthy (Original) and OSA configurations. The healthy (original) model had a velopharyngeal resistance of 54 Pa·s/L and an oropharyngeal resistance of 72 Pa·s/L. Model 4, with the severe oropharyngeal constriction, exhibited the highest regional resistance (~458 Pa·s/L) across the primitive models, while model 2, with the severe velopharyngeal constriction, exhibited a nearly similar resistance (~450 Pa·s/L). Model 6, with the severe tracheal constriction showed elevated resistance (~230 Pa·s/L) compared to the mild and original cases; however, its severity was less prominent than the oropharyngeal and velopharyngeal constrictions. Furthermore, models with multiple constrictions generally exhibited reduced regional resistance values compared to models with a single, severe constriction in a corresponding region. However, a notable exception was observed in the velopharyngeal constriction when combined with tracheal length extension (model 11), where resistance slightly increased (~460 Pa·s/L) compared to the individual velopharyngeal constriction (model 2). In fact, the resistances values computed in model 11 resemble closely those of the OSA participant, who presented a similar combination of anatomical features, specifically a severely constricted velopharynx and a comparable tracheal length. To quantify the sensitivity of regional resistance to geometric perturbations, each modified configuration was compared with the healthy geometry. Mild velopharyngeal narrowing (Model 1) increased velopharyngeal resistance by approximately 80% relative to the healthy model, whereas severe velopharyngeal narrowing (Model 2) increased it by about 730%. Mild and severe oropharyngeal constrictions (Models 3 and 4) increased oropharyngeal resistance by approximately 94% and 530%, respectively. Severe tracheal constriction (Model 6) increased local tracheal resistance by more than 2700%, but its impact on global upper-airway resistance remained lower than that of the large pharyngeal constrictions. The progression of the resistance ratio (R*) with increasing constriction severity for the primitive models is presented in Figure 6b. Resistance ratios in both the velopharyngeal and oropharyngeal regions exhibited a pronounced increase from mild to severe constriction, with the oropharyngeal region showing marginally higher resistance ratios. Conversely, the tracheal constriction had a comparatively lower influence on resistance, especially evident in the mild constriction case, which closely resembled the resistance ratio of the original model. Additionally, the OSA model displayed a remarkably high resistance ratio in the velopharyngeal region, further emphasizing the critical contribution of velopharyngeal constriction to overall airway resistance in OSA pathology. Conversely, the OSA model exhibited minimal contribution to resistance from the tracheal and oropharyngeal regions, underscoring the predominance of velopharyngeal narrowing in this participant.

### 3.3. Acoustics

**Figure 7:**
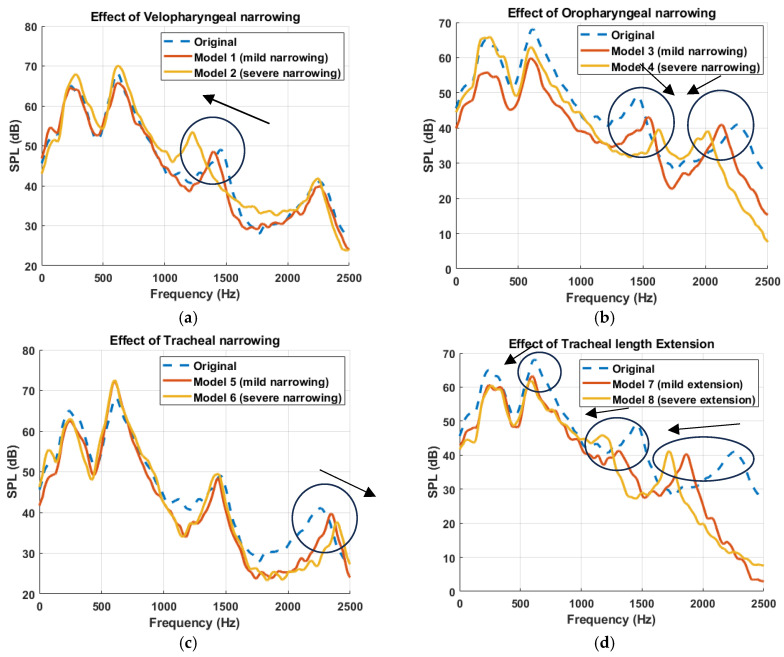
Simulated Frequency response curves at the propagation domain, downstream of the trachea, illustrating the acoustic impact of anatomical modifications to the upper airway. Each subplot compares the original airway model (dashed blue line) with two modified configurations simulating mild and severe alterations at a specific anatomical region: (**a**) velopharyngeal narrowing (Model 1: mild, Model 2: severe) [11], (**b**) oropharyngeal narrowing (Model 3: mild, Model 4: severe), (**c**) tracheal narrowing (Model 5: mild, Model 6: severe), and (**d**) tracheal length extension (Model 7: mild, Model 8: severe). Arrows have been added to indicate the direction of spectral shifts in the resonant frequencies as anatomical severity increases.

The effect of anatomical modifications on the simulated sound pressure level (SPL) is presented in Figure 7, where the frequency response curves are smoothed using a moving average filter to better visualize key spectral trends. Figure 7 compares the original (healthy) model with primitive models exhibiting mild and severe alterations in the three targeted anatomical regions. Several interesting findings emerge. The frequency response of the original model demonstrated four resonance peaks across the spectrum, serving as a baseline for comparison. These peaks were located at approximately 290 Hz, 627 Hz, 1458 Hz, and 2247 Hz. Anatomical modifications induced region-specific shifts in these resonances. For instance, as described in [11], velopharyngeal narrowing led to a progressive downward shift of the third resonance peak (1000–1700 Hz range), with increasing constriction severity (Figure 7a). In contrast, oropharyngeal narrowing caused a dual effect: the third peak (1000–1700 Hz) shifted toward higher frequencies, while the fourth peak (1700–2500 Hz) shifted toward lower frequencies, indicating a complex redistribution of spectral energy (Figure 7b). Furthermore, tracheal narrowing exhibited the least impact on the frequency response, causing only a minor shift in the fourth resonance peak (Figure 7c). This implies that compared to UA constrictions, airway modifications distal to the glottis may contribute less prominently to acoustic spectral alterations, particularly in the range critical for resonance characterization. Finally, tracheal length extension caused a downward shift in all peak frequencies (Figure 7d), consistent with the expected acoustic effects of increasing the effective resonating length of the airway [45,46].

**Figure 8:**
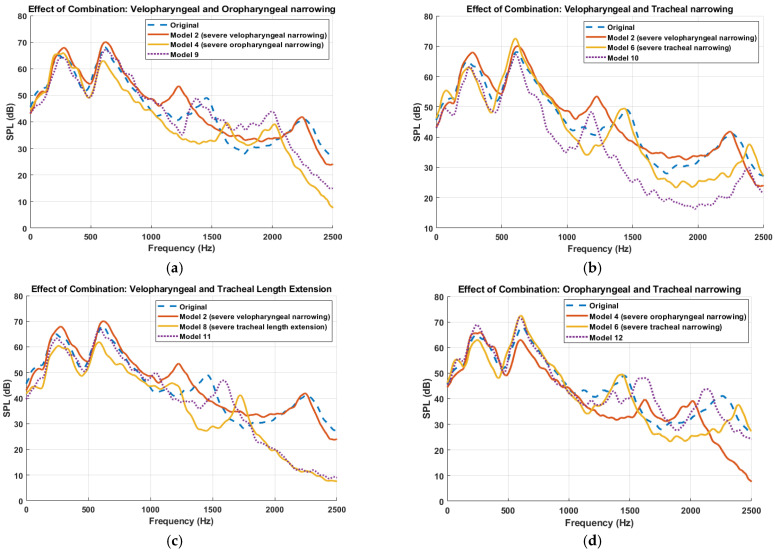

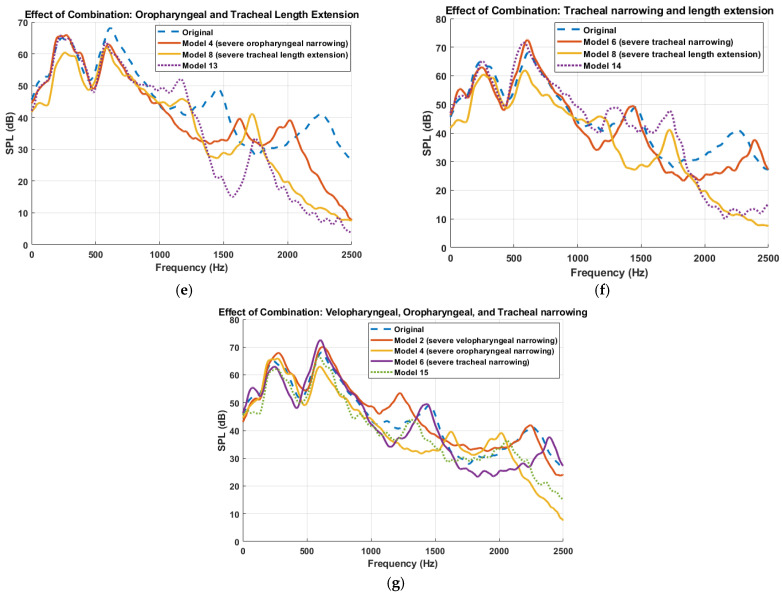
Comparison of sound pressure level (SPL) spectra for different multilevel anatomical alterations to the upper airway. Each subplot presents the frequency response for combinations of primitive anatomical modifications. (**a**) Velopharyngeal and oropharyngeal narrowing (Model 2 + Model 4 → Model 9). (**b**)Velopharyngeal and tracheal narrowing (Model 2 + Model 6 → Model 10). (**c**) Velopharyngeal narrowing and tracheal length extension (Model 2 + Model 8 → Model 11). (**d**) Oropharyngeal and tracheal narrowing (Model 4 + Model 6 → Model 12). (**e**) Oropharyngeal narrowing and tracheal length extension (Model 4 + Model 8 → Model 13). (**f**) Tracheal narrowing and tracheal length extension (Model 6 + Model 8 → Model 14). (**g**) Combined velopharyngeal, oropharyngeal, and tracheal narrowing (Model 2 + Model 4 + Model 6 → Model 15). Dotted lines represent combination models; dashed lines represent the original model. Each curve is smoothed using a moving average filter.

Furthermore, the simulated sound pressure level (SPL) spectra resulting from combined anatomical modifications to the UA is presented in Figure 8, demonstrating how multilevel constrictions interact acoustically. A key observation across all combined models is the emergence of superimposed spectral patterns, where the resulting acoustic response appears to reflect the cumulative influence of the constituent primitive models. For instance, in Model 9 (velopharyngeal and oropharyngeal narrowing), the frequency response displays both the downward shift of the third resonance peak seen in Model 2 (velopharyngeal narrowing) and the upward shift associated with Model 4 (oropharyngeal narrowing), with the effect of velopharyngeal narrowing being more pronounced, while the downward shift of the fourth resonant frequency matched the one in Model 4. This trend of spectral superposition is consistently observed across the other combination models. Model 10 (velopharyngeal and tracheal narrowing) demonstrated a downward shift in the third resonance frequency, attributed to velopharyngeal narrowing, and an upward shift in the fourth resonant frequency, consistent with the effect of tracheal narrowing. Similarly, Model 15, incorporating severe constrictions in all three regions, exhibited combined spectral effects from each anatomical modification, with the influence of the velopharyngeal constriction being most dominant, followed by oropharyngeal and then tracheal contribution. These findings suggest that, although flow–acoustic interactions within the UA are fundamentally nonlinear, particularly due to turbulence, unsteady flow separation, and geometric complexity, the system nonetheless exhibits behavior that approximates linear superposition in the frequency domain. This observation supports the notion that, under certain conditions, the spectral responses of individual anatomical modifications can be combined linearly, thereby enabling simplified modelling approaches for acoustic analysis and resonance prediction. It is important to emphasize, however, that the resonance phenomena themselves remain inherently nonlinear, governed by complex interactions between airflow, wall compliance, and geometry-dependent acoustic impedance [47].

**Figure 9:**
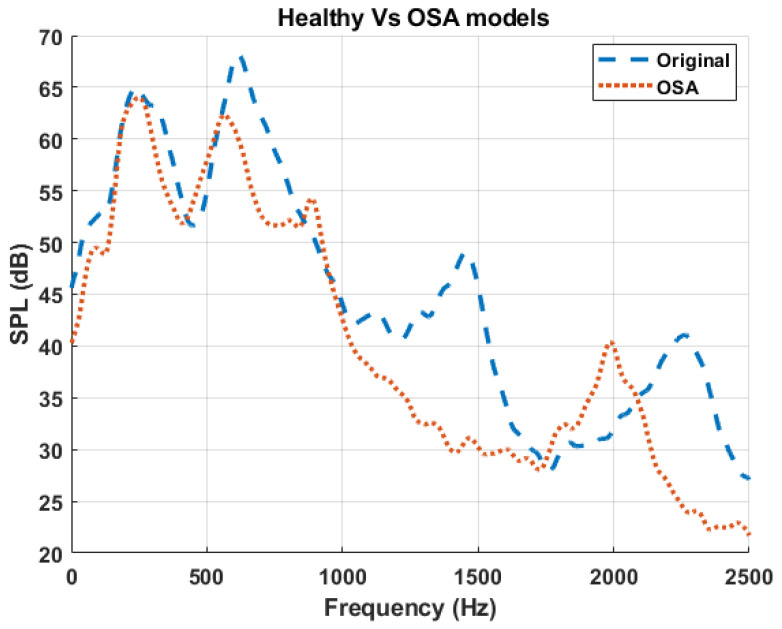
Comparison of sound pressure level (SPL) spectra between the baseline (healthy) model and the OSA participant. The dashed blue curve represents the healthy airway (Original), while the dotted red curve represents the airway geometry from the OSA participant [42].

To verify the above findings, the simulated frequency responses of both the healthy (original model) and the OSA participant are plotted in Figure 9. The OSA participant’s airway had a longer trachea and a significantly narrower velopharyngeal segment compared to that of the healthy participant. Consistent with prior observations from the primitive and combined models, the OSA spectrum demonstrated a downward shift in multiple resonance peaks, particularly the second and third peaks. This pattern aligns with the expected acoustic behavior of tracheal length extension, which lowers resonance frequencies due to an increase in the effective acoustic length of the airway, and the dominant effect of velopharyngeal constriction, which was shown to shift the third resonance peak downward. These findings further validate the hypothesis that specific anatomical changes leave distinct acoustic signatures in the frequency domain.

## 4. Discussion

This study investigated how localized and multilevel anatomical constrictions of the UA influence both aerodynamic resistance and the acoustic response of breathing sounds, with a particular focus on their relevance to the pathophysiology of obstructive sleep apnea (OSA). The findings hold potential clinical implications for the characterization and potential classification of OSA. Through systematic geometric modifications in the velopharynx, oropharynx, and trachea, we quantified the impact of these alterations on vortex formation, pressure drop, resistance distribution, and the resulting acoustic frequency response. These analyses were conducted using a non-invasive, hybrid aero-acoustic simulation framework grounded in Lighthill’s analogy and applied to realistic CT-derived UA models from both healthy and severe OSA individuals.

The findings demonstrate distinct differences in airflow behaviors between healthy versus OSA airway geometries. In the OSA model, the presence of severe velopharyngeal narrowing resulted in intensified vortex formation, characterized by dense and high-energy vortical structures downstream of the constriction. This is consistent with prior findings in vivo MRI and CFD studies that identified the velopharynx as a critical site of dynamic collapse, often associated with shear layer instabilities and high-velocity jet-like inspiratory flow. Specifically, the study in [48] reported that velopharyngeal constriction can generate a focused high-speed jet, commonly referred to as the “pharyngeal jet”, that impinges on the posterior pharyngeal wall, thereby increasing local shear forces and promoting turbulence generation. Additionally, analysis of mid-sagittal vorticity revealed that oropharyngeal narrowing (Model 4) produced the most intense and spatially concentrated vortical structures among all individual constriction models. This is noteworthy given that previous clinical studies have also identified the oropharynx as a frequent site of obstruction in OSA patients [12,49] and that oropharyngeal structure represents a significant risk factor for OSA [50]. Our results suggest that, under specific geometric configurations, severe oropharyngeal constriction may induce more pronounced flow disturbances than velopharyngeal narrowing. Interestingly, multilevel constrictions did not always result in the most severe vorticity patterns. Instead, models with a single severe constriction (e.g., Model 4) often exhibited higher localized rotational energy compared to those with distributed moderate constrictions (e.g., Model 9). This suggests that singular anatomical bottlenecks may dominate flow disruption, emphasizing the importance of site-specific anatomical evaluation in clinical settings.

Regional airway resistance was computed for each anatomical segment to evaluate the localized impact of each structural modification. Previous studies have established the role of airway resistance as a biomarker for OSA severity classification. For instance, OSA patients were reported to have airway resistance values approximately three times higher than those of healthy controls, with mean resistances of 1173 Pa·s/L and 374 Pa·s/L, respectively [51]. Strong correlations between pharyngeal airway resistance and the apnea-hypopnea index (AHI) have also been reported [52,53]. Furthermore, interventions such as oral appliances have been shown to reduce airway resistance by up to 36% in OSA patients [54]. In this study, we investigated the specific impact of anatomical constriction at each segment on the resistance of each anatomical site and its relative on the overall airway resistance. Dimensionless resistance ratios (R*) further illustrated the differential contribution of each anatomical segment to total airway resistance. In both primitive and combined models, velopharyngeal and oropharyngeal segments were the primary contributors to global resistance. In contrast, tracheal constriction, even when severe, exerted a comparatively smaller influence. Notably, Model 11, which combined velopharyngeal constriction with tracheal length extension, resulted in a higher velopharyngeal resistance than the model incorporating velopharyngeal narrowing only. The resistance profile closely resembles that of the OSA participant’s geometry. This supports previous findings [55], who demonstrated that UA lengthening, is a structural characteristic of OSA and correlates strongly with AHI. While their study focused on morphological changes and their relationship with AHI, our results extend this understanding by showing that airway elongation can also act with UA narrowing to exacerbate aerodynamic resistance, thereby contributing to the airflow limitation observed in OSA.

Furthermore, the impact of the anatomical modifications on the simulated TBS were studied. The frequency response analysis of the UA models revealed region-specific spectral shifts corresponding to anatomical modifications. In particular, velopharyngeal narrowing produced a progressive downward shift in the third resonance peak (1000–1700 Hz range) with increasing severity [11], while oropharyngeal narrowing resulted in a dual pattern: an upward shift of the third peak and a downward shift of the fourth. These patterns reflect underlying changes in the acoustic properties of the airway segments and can be interpreted using the principles of acoustic inertance (or acoustic mass) and compliance. In a simplified lumped acoustic system, the resonant frequency of a given segment is determined by the balance between its acoustic compliance (C) and acoustic mass (M) [47], described by

(2) $$f_{r}=\frac{1}{2\pi }\sqrt{\frac{1}{MC}}$$

where acoustic mass (M) is given by

(3) $$M=\frac{\rho L}{A}$$

and acoustic compliance (C) is defined as

(4) $$C=\frac{V}{\rho c^{2}}$$

Here, V is the volume, L is the effective length of the segment, A is the cross-sectional area perpendicular to the air column acceleration, ρ is air density, and c is the speed of sound.

In the velopharyngeal constriction models, the narrowing reduces the cross-sectional area A, increasing acoustic mass (M), which in turn lowers the resonant frequency ($f_{r}$) explaining the observed downward shift of the third peak. Although the segment volume also decreased, reducing acoustic compliance, the effect of reduced area on acoustic mass was more pronounced. This suggests that the constricted velopharynx behaves as a region dominated by acoustic mass, acting like a bottleneck that delays pressure wave propagation and reduces the system’s natural frequency in the [1000–1700] Hz range. Conversely, in oropharyngeal constriction, the third peak shifted upward while the fourth shifted downward. The upward shift is likely due to a reduction in compliance, i.e., reduced oropharyngeal volume V, leading to increased stiffness and a higher $f_{r}$. The downward shift of the fourth peak may reflect increased acoustic inertance effects, which become more influential at higher frequencies. Since acoustic compliance and inertance are both frequency-dependent [47], their relative influence shifts across the spectrum. In this case, the increased influence of acoustic inertance of the airway at higher frequencies likely contributed to the selective lowering of the fourth resonance peak. This complexity suggests that oropharyngeal geometry influences both local and distributed acoustic behaviors, modulating the airway’s filtering characteristics in a frequency-dependent manner. This highlights the potential of resonance-based analysis as a tool for inferring site-specific airway alterations from non-invasive tracheal sound measurements, with direct implications for OSA diagnosis.

This study has potential clinical implications. The findings suggest that patient-specific airway models could serve as valuable tools for non-invasive functional assessment of airway mechanics, particularly when combined with tracheal breathing sound (TBS) analysis. The observed spectral shifts in TBS, which correspond to distinct anatomical modifications, support the feasibility of using acoustic signatures as indirect markers of localized airway narrowing or elongation. This could open the door to sound-based screening tools that leverage computational modelling to interpret patient-recorded TBS data in terms of site-specific anatomical abnormalities. Such methods may reduce reliance on full polysomnography and provide accessible and cost-effective screening pathways, particularly in primary care or home-based monitoring settings. Moreover, the demonstrated ability of the model to capture how multilevel constrictions influence spectral patterns may facilitate personalized treatment planning, such as selecting optimal sites for surgical intervention or designing patient-specific oral appliances. Ultimately, this integrative modelling and acoustic approach could complement existing diagnostic methods by providing mechanistic insight into the airflow acoustic interactions underlying OSA pathophysiology. In practical terms, future TBS-based diagnostic algorithms could incorporate simulation-informed spectral features together with anthropometric and clinical variables in multivariate or machine-learning models to estimate OSA probability and localize the obstruction site. Future work should develop a mathematical formulation of the relationship between airway anatomy and the breathing sounds spectrum using parameterized models and develop algorithms suitable for clinical and home devices, improving the robustness, interpretability, and clinical utility of TBS-based screening tools.

## 5. Limitations

The limitations of this study include the assumption of rigid airway walls, which did not incorporate fluid–structure interactions during inspiration. The absence of wall compliance neglects potential tissue deformation and collapsibility, particularly in regions prone to obstruction such as the velopharynx and oropharynx. CFD boundary conditions included a fixed pressure drop to simulate inspiratory flow. While this approach facilitates comparison across anatomical models, it does not fully capture the transient pressure fluctuations characteristic of natural breathing. In particular, prescribing a single steady pressure drop neglects the acceleration and deceleration phases of inspiration and inter-subject differences in driving pressure associated with lung volume or breathing effort level. Nevertheless, this is justified by the fact that the aero-acoustic model was validated [11] using tracheal breathing sounds recorded during the plateau phase of inspiration, where flow is relatively stable. Accordingly, the absolute resistance and TBS amplitudes reported here should be interpreted as corresponding to a representative inspiratory effort, rather than to the full dynamic range of breathing conditions.

Another important limitation is the restricted participant representation. This study focused on a single healthy subject and one OSA patient, both serving as representative anatomical cases. However, the healthy participant was 76 years old, while the OSA participant was 47 years old, introducing an age-related anatomical variability that may confound the interpretation of disease-specific findings. Aging affects the UA anatomy through changes in muscle tone, tissue compliance, and airway geometry [56,57]. Although the primary aim of this study was to explore anatomical contributions to acoustic response independent of demographics, the age disparity may still affect the UA shape and resonance characteristics. Therefore, future studies should account for age-matched controls or include larger cohorts with balanced age, sex, and BMI distributions to isolate pathology-specific effects.

Furthermore, while the anatomical modifications applied to the healthy model were guided by clinical imaging data, the study did not explore the full spectrum of OSA anatomical variability, severity levels, or sex-based differences in the UA structure. Future work should include a larger cohort of OSA patients with diverse anatomical phenotypes to improve the generalizability of the findings and strengthen their clinical relevance.

## 6. Conclusions

This study investigated how specific anatomical features of the upper airway influence airflow dynamics and tracheal breathing sounds spectra using one-way coupled aero-acoustic modelling framework. By systematically introducing controlled velopharyngeal, oropharyngeal and tracheal constrictions, as well as the airway elongation, the contribution of each anatomical modification to the airway resistance and acoustic response was examined. The acoustic analysis showed that each anatomical region imprints a distinct and interpretable signature on the TBS frequency response. Velopharyngeal narrowing caused a downward shift of the third resonance peak, oropharyngeal narrowing produced opposite shifts of the third and fourth peaks, tracheal narrowing had a comparatively mild effect, and tracheal elongation lowered all resonances by increasing the effective acoustic length. Multilevel constrictions yielded spectra that closely resembled a superposition of the corresponding primitive responses. These findings establish a mechanistic link between localized anatomical changes and TBS acoustic response, supporting the use of anatomically informed spectral features as potential non-invasive markers of site-specific airway pathology in OSA.

## Figures and Tables

**Table 1:** Summary of anatomical modifications applied to the CT-based healthy airway model. Primitive models represent isolated modifications to individual anatomical regions, while combination models incorporate multilevel alterations.

	Model ID	Target Region			Constriction Level		Length Extension Level		Variation from Original Model			
		VP	OP	Tr	Mild	Severe	Mild	Severe	Min CSA	%	Length	%
Primitive models	Model 1	†**			†				85 → 62 mm^2^	−27%		
	Model 2	†				†			85 → 26 mm^2^	−69%		
	Model 3		†		†				190 → 85 mm^2^	−55%		
	Model 4		†			†			190 → 30 mm^2^	−84%		
	Model 5			†	†				166 → 67 mm^2^	−60%		
	Model 6			†		†			166 → 36 mm^2^	−78%		
	Model 7			†			†				135 → 155 mm	15%
	Model 8			†				†			135→ 175 mm	30%
Combinations	Model 9	†	†			††			Model 2 + Model 4			
	Model 10	†		†		††			Model 2 + Model 6			
	Model 11	†		†				†	Model 2 + Model 8			
	Model 12		†	†		††			Model 4 + Model 6			
	Model 13		†	†		†		†	Model 4 + Model 8			
	Model 14			†		†		†	Model 6 + Model 8			
	Model 15	†	†	†		†††			Model 2 + Model 4 + Model 6			

** † = constriction in a single target region; †† = constriction in two regions; ††† = constriction in three regions. VP: Velopharynx; OP: Oropharynx; Tr: Trachea; Min CSA: minimum cross-sectional area.

**Table 2:** Volumetric and cross-sectional area (CSA) measurements for each targeted upper airway region (velopharynx, oropharynx, trachea) across the healthy baseline, individual modification models, and the OSA geometry. For each region, both the segmented volume (in mm^3^) and the minimum cross-sectional area (in mm^2^) are listed.

Model	Velopharynx		Oropharynx		Trachea	
	Volume (mm^3^)	Min CSA (mm^2^)	Volume (mm^3^)	Min CSA (mm^2^)	Volume (mm^3^)	Min CSA (mm^2^)
Healthy Original	2616	85	6009	190	6517	166
Model 1	2291	62				
Model 2	2031	26				
Model 3			4798	85		
Model 4			3683	30		
Model 5					5859	67
Model 6					5530	36
OSA	1540	28	6003	110	11,373	255

## Data Availability

The data presented in this study are available on request from the corresponding author due to privacy and ethical restrictions.
